# Reconstruction of a massive facial defect with the pre-expanded free anterolateral thigh flap: A case report

**DOI:** 10.1016/j.ijscr.2021.106693

**Published:** 2021-12-27

**Authors:** Tran Thiet Son, Pham Thi Viet Dung, Le Anh Huy

**Affiliations:** a Department of Plastic Reconstructive and Aesthetic Surgery, Bach Mai Hospital, No.78 Giai Phong Street, Hanoi, Viet Nam; b Department of Plastic Surgery, Saint Paul Hospital, No.78 Giai Phong Street, Hanoi, Viet Nam; c Department of Plastic and Reconstructive Surgery, Hanoi Medical University Hospital, No.78 Giai Phong Street, Hanoi, Viet Nam

**Keywords:** Pre-expansion, Free ALT flap, Secondary thinning, Massive facial defect, Reconstruction

## Abstract

**Introduction and importance:**

Massive facial defects remain a reconstructive challenge because of the region's unique character and the limitation of a well-matched donor site. Pre-expanded free anterolateral thigh (ALT) flap provides a good alternative for reconstructing massive skin and soft tissue defects.

**Case presentation:**

A 21-year-old male patient presented to our department with a massive right facial degloving wound caused by a sharp edged-weapon attack. The patient left the hospital after 3 weeks of wound care, and only came back to us after 2 months with large defects at the nasal, cheek, and upper and lower right lip regions. The procedure of ALT flap expansion surgery was performed for 2 months. The pre-expanded ALT flap was used for reconstruction of the patient's facial defects. Two-year follow-up showed that the flap at the reconstructed area resembled the contour of the nasal tip, facial skin color similarities in the cheeks and lips, and the patient's mouth had normal function.

**Clinical discussion:**

The combined pre-expanded and composite ALT free flap techniques allow simultaneous reconstruction of many different anatomic units. Flap debulking helps to improve the nasal contour from the original defect. The lip separation technique and flap debulking procedure help enhance aesthetic reconstructive outcomes of the skin flap.

**Conclusion:**

The surgical reconstruction using a pre-expanded ALT free flap for the patient with a massive facial defect was safe. In particular, sufficient skin and soft tissue ensured facial aesthetics and oral function for the patient when using this method.

## Highlights

• Pre-expanded anterolateral thigh flap provides an alternative skin for reconstruction.
• A 21-year-old male patient with a massive facial defect.
• The reconstruction was safe and restored facial aesthetics and function

## 1 Introduction

Massive facial defects occur from injuries caused by gun blast injury, burns, or oncological resection. They often lead to large facial deformities and disfigurement, which remains a reconstructive challenge. Expanded local flaps can produce a sustainable solution for the reconstruction of medium facial defects, but in case of damaged local tissues, distant tissue free flap transfer is the optimal choice [1]. One of the most popular approaches has recently been to use the anterolateral thigh (ALT) flap because of its constant, reliable anatomy and various clinical applications [2], [3]. In addition, tissue expansion is also an approach to increasing skin and soft tissue for defect closure. A combination of tissue expansion and a free ALT flap was introduced for resurfacing the large territory, such as in postburn neck scar contracture [4], [5]. Tissue expansion placement at the subfascial or suprafascial ALT flaps is performed in reconstructing large burn scars [6], [7]. This technique is a true pre-expanded ALT flap because it provides enough skin surface area to close the donor site, and changes the structure of the microsurgical flap. This report presents a successful reconstruction for a patient with a massive facial defect using the pre-expanded free ALT flap. This report has been written in accordance with SCARE guidelines criteria [8].

## 2 Presentation of case

**Fig. 1:**
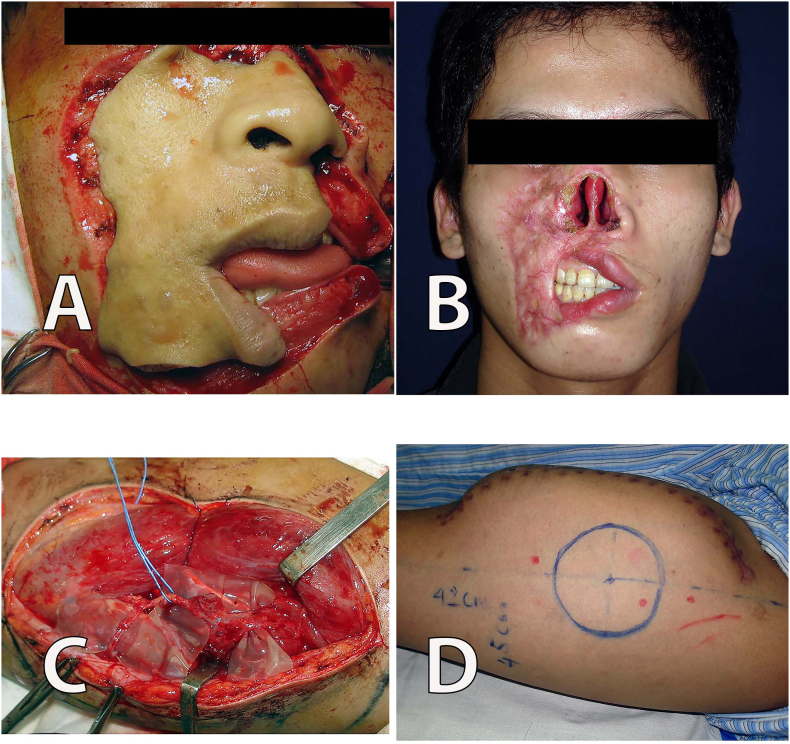
A. Facial view at the time of initial visit; B. Facial view with scars and soft-tissue defects after 3 months of accident; C. Dissection of the anterolateral thigh flap to insert the tissue expander; D. The area of anterolateral thigh with the tissue expander after 2 months (at the end of expansion period).

A 21-year old male patient was admitted to our hospital in critical condition with a tearing injury caused by a sharp-edged weapon on the right side of the face (Fig. 1A). The patient was operated on, but the replantation failed because the arteries and veins of the avulsed tissues were too small. The degloving skin flap was removed on the following day, and only the skin was kept to cover the wound. The patient then underwent wound care for 3 weeks. The patient left the hospital, and only returned to our hospital 2 months later with scarred soft-tissue defect of the full nasal area, the right labial area, and the right cheek (Fig. 1B). He showed a large defect of the total nasal tip, nasal dorsum, sidewalls, alae, and columella with the exposed nasal septum, and the right-sided upper and lower lip vermillion. The patient was unable to close his mouth, and had ongoing salivation.

We advised the patient of several surgical options based on the patient's condition, but the patient would like to have the surgery done only once. Thus, we decided to use the anterolateral thigh (ALT) and tissue expander for reconstruction. We used a handheld Doppler to identify perforators of the descending branch of the lateral circumflex femoral artery. A 12-cm horizontal incision was made on the lateral thigh. Then, two perforators entering the flap were identified, and these perforators were separated as descending branches from the vastus lateralis muscle. A silicone tube was used to wrap around the perforator and descending branches to determine the vascular pathway (Fig. 1C). A 380-mL rectangular tissue expander was placed under the flap, and the closure process was performed in a three-layer fashion. The expansion was initiated from the 7th day, and it was continued for 2 months to reach a volume of 1208 mL (Fig. 1D).

**Fig. 2:**
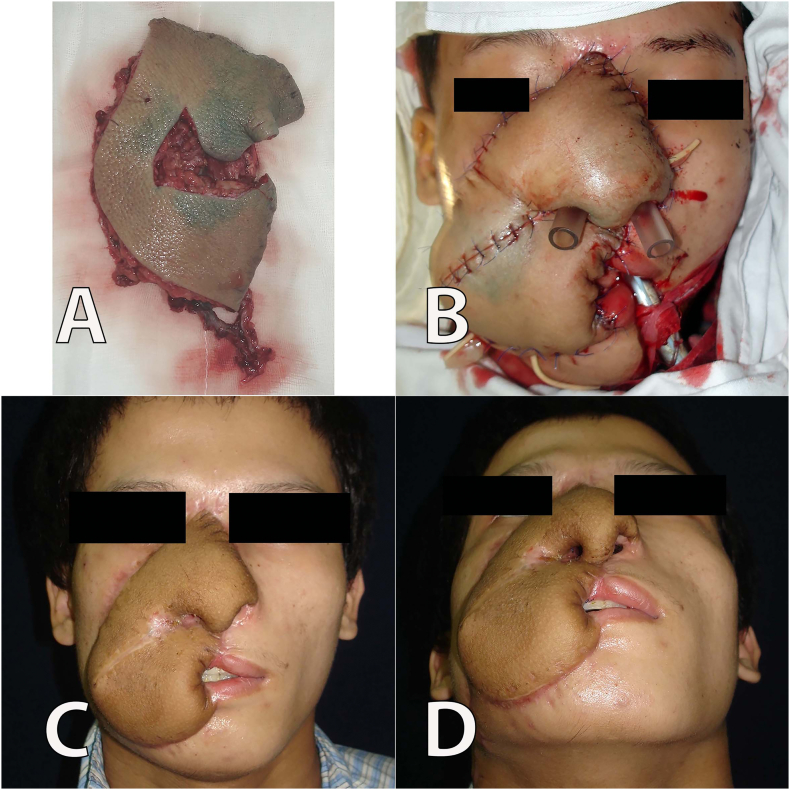
A. The anterolateral thigh skin flap measured 20 × 29 cm was harvested, it was then divided into two parts to shape the nose and lips; B. Shows the patient's face after the surgery; C, D. Face view 1 month after the surgery.

**Fig. 3:**
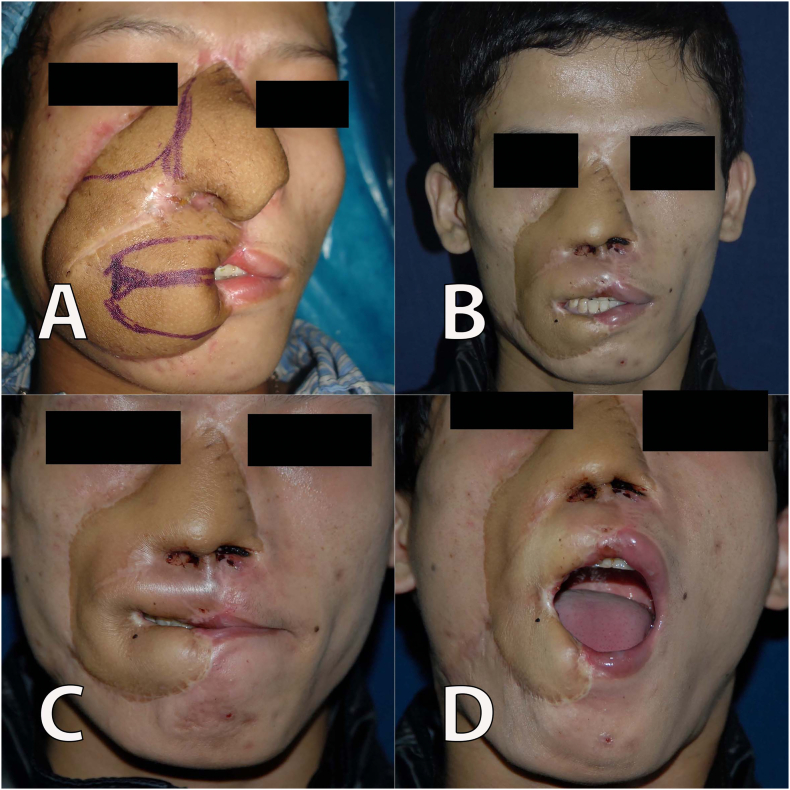
A. Before dissecting the flap to create the contour of the upper and lower lip vermilion; B. face view after 2 years; C and D: The patient was able to open and close his mouth.

The size of the soft-tissue defect was estimated at 15 × 18 cm. We prepared the right facial artery as the recipient's vessel. A piece of the iliac crest bone with the size of 2 × 5 cm was harvested, and the bone framework was fixed to the maxilla using a titanium micro-plate and screws. We used rib cartilages to reconstruct the two alar cartilage. A new incision was made over the previous one to remove the tissue expander. Two perforators and the descending branch were identified, and the capsule was removed. A 20 × 29 cm flap, of which 70% was from the expanded skin, was elevated. We harvested the entire expanded tissue to maximize flap size. In addition, we only collected the minimum amount of tissue in the medial area. Two perforators from the same source vessel, and a 14 cm-in-length descending branch from the anterior circumflex femoral artery were harvested (Fig. 2A). Two skin flaps were designed to ensure that each flap was supplied by one perforator vessel and both skin flaps were adjacent to the other. A perforator flap was used for the nasal defect reconstruction with the addition of flap thinning to create contours of the nose, columella, and nasal inner lining. Meanwhile, the other perforator flap was used to reconstruct the right upper and lower lip vermilion and check defects. The flap was transferred to the face and the artery and vein were anastomosed to the facial artery and vein in an end-to-end fashion (Fig. 2B). The donor area was mostly closed primarily and a skin graft was used with the remaining defect measuring 3 × 5 cm. The flaps were well vascularized at the recipient site and survived without morbidity (Fig. 2C, D). The patient received Heparin 1000UI within 24 h of surgery. After one month, the patient underwent an operation to separate the flap into a lip-like structure and create the oral commissure (Fig. 3A). We placed silicone tubes in the patient's nostrils for two months. Furthermore, since we performed the lining, the patient's nasal cavity was wide enough for him to take care of himself. There was no problem with the retraction of the mucosa of the nose. The skin flaps at the nasal and cheek areas were thinned twice within 6 months postoperatively. After 2 years of follow-up, the flap at the reconstructed area resembled the contour of the nasal tip (Fig. 3B). The patient was able to open and close his mouth with the normal range (Fig. 3C, D).

## 3 Discussion

The free ALT flap has become popular in recent years with its versatility in reconstruction for defects of different body parts, including facial defects [2], [9]. The free ALT flap is considered to have many advantages with surgical reconstruction because it has reliable vascularity and large cutaneous territory. Most importantly, the ALT flap is easily harvested, and minimizes donor site deformity. However, the conventional ALT flap is often too bulky for resurfacing face defects, so it often needs to be performed a secondary debulking procedure. Moreover, the disadvantages of the free ALT are that skin color and texture are not similar to facial skin, and there is a high incidence of hair presence, especially in male patients [10], [11], [12], [13].

Since the first description of a pre-expanded sensate lateral arm free flap by Shenaq et al. in 1987 [14], many donor sites have been applied with this technique for various indications. Hallock et al. used tissue expansion by placing it sub-facially laterally to the ALT flap design mainly for the donor-site closure [4], [5]. Meanwhile, tissue expansion was used by Hocaoglu et al. for closure of the donor site after the ALT free flap was harvested and used for reconstruction of hand defects, but the expanded area was not transferred [15], [16]. Tsai et al. first used pre-expanded flap as an innovative technique in reconstruction after the release of burn scar contracture, precluding the advantages created by the expansion process, like thinning the tissue for better skin resurfacing and increase in vascularity [7]. Acarturk et al. also used the pre-expanded ALT free flap for reconstruction of postburn cervical scar contracture. He placed a tissue expander underneath the ALT flap and hence, the obtained ALT flap accounted 90% of the pre-expanded flap [17], [18]. Most importantly, a pre-expanded perforator flap offers an effective option for reconstruction with a well-vascularized flap.

In this case report, we used the ALT flap to reconstruct for the patient with the large facial soft tissue defect caused by the post-traumatic scar lesion. Although the paramedian forehead flap was recommended for nasal reconstruction, the patient refused this approach because he did not want to have more facial scars. In addition, tissue expansion at the right cervicofacial area could be applied to cheek and lip defects. However, if applied in this approach, the patient would have to undergo multiple surgeries. Therefore, the pre-expanded ALT free flap provided the most reliable option in this case. In a previous study, Acarturk et al. suprafascially placed the expander 2 cm laterally to the pre-localized perforator sites [17]. Meanwhile, we placed the expander between the muscle and fascia, similar to the technique described by Tsai et al. [7]. Unlike the perforator dissection performed by the two mentioned authors, the dissection in our case was carried out to determine the course of required perforators, descending branch, and potential anatomical variations. We wrapped the vessels with a silicone tube for dual purposes, protecting the arteries during tissue expander filling and facilitating the pre-expanded ALT flap harvesting stage. The tissue expander was placed underneath the proposed flap and perforators, which helped the pre-expanded ALT flap fully stretch and slowly increase the flap survival rate. As a result of using the silicone tube, the vascular pedicle was easily identified and dissected. The pre-expanded flap was divided into two parts, each of which was vascularized by a perforator. To reconstruct the cheek structure, two skin flaps were connected by a skin bridge. A part of the flap was employed for lip defect reconstruction, which later was split to create lip-like structures. The remaining flap, as being too thick (1.8 cm), was thinned down to 8 mm, and then was used to recreate nasal subunits and inner lining.

## 4 Conclusion

The surgical reconstruction using a pre-expanded ALT free flap for the patient with a massive facial defect was safe, and restored the patient's facial aesthetics and oral function. This method could be the foundation to apply to patients with massive facial defects in the future. In addition, we also found that performing flap debulking can help improve the results of facial reconstruction.

## Abbreviation

ALT: anterolateral thigh
FL: fascia lata

## Sources of funding

No source to be stated.

## Ethical approval

Approval is not necessary for a case report in our locality.

## Consent

Written informed consent was obtained from the patient for publication of this case report and accompanying images. A copy of the written consent is available for review by the Editor-in-Chief of this journal on request.

## Author contribution

Tran Thiet Son: first and corresponding author, performed the operation, conceptualization, writing and revising the manuscript.

Pham Thi Viet Dung: writing and revising the manuscript.

Le Anh Huy: writing and revising the manuscript.

## Registration of research studies

N/A.

## Guarantor

Tran Thiet Son. Ph.D. M.D.

## Provenance and peer review

Not commissioned, externally peer-reviewed.

## Declaration of competing interest

Authors do not report any conflict of interest.
